# Explore the Features of Brain-Derived Neurotrophic Factor in Mood Disorders

**DOI:** 10.1371/journal.pone.0128605

**Published:** 2015-06-19

**Authors:** Fan-Chi Yeh, Chung-Feng Kao, Po-Hsiu Kuo

**Affiliations:** 1 Department of Public Health & Institute of Epidemiology and Preventive Medicine, College of Public Health, National Taiwan University, Taipei, Taiwan; 2 Research Center for Genes, Environment and Human Health, National Taiwan University, Taipei, Taiwan; University of Illinois at Chicago, UNITED STATES

## Abstract

**Objectives:**

Brain-derived neurotrophic factor (BDNF) plays important roles in neuronal survival and differentiation; however, the effects of BDNF on mood disorders remain unclear. We investigated *BDNF* from the perspective of various aspects of systems biology, including its molecular evolution, genomic studies, protein functions, and pathway analysis.

**Methods:**

We conducted analyses examining sequences, multiple alignments, phylogenetic trees and positive selection across 12 species and several human populations. We summarized the results of previous genomic and functional studies of pro-BDNF and mature-BDNF (m-BDNF) found in a literature review. We identified proteins that interact with *BDNF* and performed pathway-based analysis using large genome-wide association (GWA) datasets obtained for mood disorders.

**Results:**

BDNF is encoded by a highly conserved gene. The chordate *BDNF* genes exhibit an average of 75% identity with the human gene, while vertebrate orthologues are 85.9%-100% identical to human *BDNF*. No signs of recent positive selection were found. Associations between *BDNF* and mood disorders were not significant in most of the genomic studies (e.g., linkage, association, gene expression, GWA), while relationships between serum/plasma BDNF level and mood disorders were consistently reported. Pro-BDNF is important in the response to stress; the literature review suggests the necessity of studying both pro- and m-BDNF with regard to mood disorders. In addition to conventional pathway analysis, we further considered proteins that interact with BDNF (I-Genes) and identified several biological pathways involved with BDNF or I-Genes to be significantly associated with mood disorders.

**Conclusions:**

Systematically examining the features and biological pathways of BDNF may provide opportunities to deepen our understanding of the mechanisms underlying mood disorders.

## Introduction

Brain-derived neurotrophic factor (BDNF) is a member of the family of neurotrophic factors. It was first explored and purified from pig brain in 1982, and other members of the neurotrophin family were successively discovered [1]. In humans, the BDNF protein is first released to the Golgi apparatus in longer precursor forms (pro-BDNF), which are then cut by specific enzymes to obtain mature BDNF (m-BDNF) [2]. The m-BDNF and pro-BDNF forms have active but opposite functions in biological mechanisms. The function of m-BDNF is related to neuron survival, differentiation, and development through binding to tropomyosin-related kinase B (TrkB) receptor to induce a series of downstream pathways [3]. In contrast, Pro-BDNF binds to p75 neurotrophin receptor to induce cell apoptosis and long-term depression [1, 4, 5]. Because of their importance in maintaining the growth, survival, and function of neurons, exploring the potential roles of *BDNF* in brain disorders has been a long-standing focus, especially for mood disorders [6].

In past decades, various study approaches have been undertaken to explore the functions and associations of *BDNF* with mood disorders, such as assessing gene expression or the serum BDNF level, conducting linkage studies, or conducting individual and genome-wide association (GWA) studies. Inconsistent findings are often observed across or even within specific study designs. It is unlikely to achieve a comprehensive evaluation without combining information from different sources. Thus, exploration of the relationships between *BDNF* and mood disorders could benefit substantially from a systematic review of the currently available evidence from different study designs. Recently, more innovative approaches have been adopted to consider the complexity of molecular actions underlying complex traits, such as considering protein-protein interaction (PPI) or conducting pathway-based analyses. There have been some successful applications to psychiatric traits, such as schizophrenia and major depressive disorder [7–10]. For instance, candidate genes of schizophrenia are found to share common PPIs and act on the same biological processes [11]. Thus, it is essential to adopt strategies from systems biology and to incorporate PPI information and pathway analysis to enhance our understanding of the roles of *BDNF* in mood disorders.

Although a variety of genetic epidemiology designs have been used to study *BDNF*, other works focusing on its gene features, molecular evolution, and protein interactions with other molecules remain scarce and understudied. The adaptive selection and phylogenesis of the *BDNF* gene in 35 organisms have been previously examined in vertebrates [12]. However, the magnitude of natural selection in higher organisms and human beings requires further study. In this study, we adopted the systems biology perspective and applied several approaches to explore the features of *BDNF* in relation to mood disorders. We first evaluated its sequence conservation among species and the magnitude of positive selection in different human populations. Second, we reviewed curated evidence from a systematic review and meta-analysis of different study designs to evaluate the associations between *BDNF* and mood disorders, as well as evidence from basic experimental designs for the roles of pro-BDNF in emotion related traits. Finally, we investigated the protein interactome of BDNF and applied pathway-based analysis to examine the enrichment of biological pathways involved with BDNF in major depressive disorder (MDD) and bipolar disorder (BPD) separately, using two large GWA datasets.

## Materials and Methods

### 1. Molecular evolution

#### Gene structure, sequence identity, and multiple alignment

We compared 12 species in vertebrates, including 8 mammals (human, chimpanzee, macaque, dog, pig, cattle, mouse and rat) and 4 non-mammals (finch, turkey, chicken and zebrafish). We first searched the *BDNF* gene sequence, coding DNA sequence (CDS), and proteins in the NCBI Entrez Gene and Ensembl databases. For species with multiple transcripts in the database, the longest one was selected to include the most information. We used RepeatMasker (open version 3.3.0, http://www.repeatmasker.org/), a program to screen interspersed and low complexity DNA sequences, to identify different types of repeat sequence to analyze the underlying structure of the genes. The ABBlast searching engine was applied in this program for the advantages of time-saving and good sensitivity.

To compare the similarity between humans and the other species, we used the Basic Local Alignment Search Tool (BLAST) suites to calculate pairwise sequence identity rate for both the CDS nucleotides (blastn suite) and the amino acids of the protein sequence (blastp suite). BLAST is widely used in the comparison of query nucleotides or amino acid sequences and its sequence database, which calculates the similarity statistical significance by the Expected value (E value). The E value threshold was set to 10 by default, indicating a match number of 10 with a sequence of the same length in the database by chance. When the E value was smaller than 10, the pairwise identity and similarity significance were tested among species in BLAST. Multiple sequence alignment of the protein across species was performed by ClustalX (version 2.0) [13], illustrating sequence conservation. Finally, to evaluate the evolutionary relationships and distances between species, we used MEGA 5 to construct phylogenetic trees by neighbor-joining methods [14].

#### Estimation of positive selection

The detection of adaptive selection across species was performed by PAL2NAL (version 14) [15]. The BDNF protein sequence and coding region DNA sequence were input into PAL2NAL. These sequences were converted into codons, and the ratio of the non-synonymous over the synonymous substitution rate (dN/dS) was pair-wise calculated among species using the codeml program in the Phylogenetic Analysis by Maximum Likelihood package. As most non-synonymous substitution is expected to occur under purifying selection, the gene is considered to experience positive selection when the dN/dS ratio is greater than 1. We also used Happlotter [16] to examine whether the *BDNF* gene has undergone recent positive selection during evolution in humans. This web application uses the HapMap phase II (release 21) allelic frequency data of three populations in its analysis, including Utah residents with northern and western European ancestry from the CEPH collection (CEU), Yoruban in Ibadan, Nigeria (YRI), and East Asians (ASN). The measurements of positive selection are calculated by the Integrated Haplotype Score (iHS), which is based on the difference in linkage disequilibrium between a selected and a background allele at the same position. Positive selection is considered to be significant when the iHS is higher than 2 [16].

### 2. Curated evidence from systematic literature review

Due to the great interest in studying *BDNF* and its protein in the context of mood disorders, a substantial amount of data has been accumulated. We performed a literature review by searching in the PubMed database for two topics: genomic studies of *BDNF* in humans and biological functions of pro-BDNF regardless of species. For the first topic, a number of meta-analysis and review articles have been published in the past decade. Articles were limited to English-language, full text, publication year between 2003 and 2013, human species, and review or meta-analysis as the article type. We also reviewed some of the references of these original articles when necessary. For the second topic, we focused on the biological functions of pro-BDNF in relation to emotion. Article types and species were not restricted. The search in the literature for mood disorder and emotion-related keywords used several MeSH terms, including mood disorder, major affective disorder, major depressive disorder, depression, bipolar disorder, mania, or stress. All the articles initially found in the systematic literature review process were manually checked for their relevance to our topics.

There are different designs in genomic studies of *BDNF* in human, and we divided these studies into categories of gene expression, linkage, genetic association and GWA studies, and serum/plasma BDNF level. A special note is for gene expression studies. Gene expression data and analysis results are affected by tissue types, experimental platforms, and characteristics of samples. Some gene expression results are deposited in the SMRI (Stanley Medical Research Institute), which collected brain tissues of patients with schizophrenia and bipolar disorder. Detailed experimental data of gene expression from12 studies were obtained from the SMRI database. In addition to literature review, we extracted gene expression data from the SMRI database to perform direct analysis, which provided the consensus fold change by weighted combination of individual probes mapping to genes across studies.

### 3. Protein interactome and pathway analysis

#### Protein-protein interaction (PPI)

To identify proteins that interact with BDNF, we used the STRING database (Search Tool for the Retrieval of Interacting Genes/Proteins, version 9.0). STRING includes more than 5 million known and predicted PPI from 1,133 organisms. It also provides an evidence score (ranging between 0 and 1) for the prediction of PPI from a variety of data sources, including experimental data on physical PPIs, annotated pathway sources, organism-specific or text-mining databases, and predicted associations via gene neighborhoods, fusion, co-occurrence, and co-expression. The higher the evidence score is, the greater is the confidence that the interaction exists. A molecule with an evidence score greater than 0.4 was considered to have medium confidence of interaction with BDNF, according to the suggestion of this web tool. Genes encoding proteins that interact with BDNF were named I-Genes in our study. Using the prediction results of STRING, we collected a total of 363 I-Genes based on predicted interactions with BDNF. We also plotted the PPI network of I-Genes with evidence scores greater than 0.9 in S1 Fig for easy visualization.

#### Pathway annotation and genome-wide association datasets

We downloaded canonical pathway information from the Molecular Signatures Database (MSigDB, version 3.0) for pathway annotation. The MsigDB is a large integrated database from several available pathway sources, in which we focused on two main collections, the manually curated pathways based on expert knowledge and online pathway databases such as the Kyoto Encyclopedia of Genes and Genomes, BioCarta, Reactome, and Gene Ontology. In total, 4726 pathways were annotated. We further selected two sets of pathways to be included in our analysis for BPD and MDD: the *BDNF* gene-involving pathways (named BDNF-pw, see S2 Table.) and the *BDNF* PPI-related pathways that include I-Genes in the gene-set (named IGene-pw, see S2 Table.). The definition of IGene-pw is to have more than 10% I-Genes in the total gene number of a pathway. In total, there were 45 and 37 pathways for BDNF-pw and IGene-pw, respectively. Among them, two pathways overlapped. Pathways with extreme numbers of genes, i.e., more than 380 or less than 10 (i.e., the 10th percentile of pathway size distribution) were excluded from analysis to avoid stochastic bias or overly general biological processes. Finally, we included 29 pathways in BDNF-pw (consisting of 3,265 genes) and 34 in IGene-pw (consisting of 1,818 genes) in the following pathway analysis.

The GWA datasets of MDD and BPD for pathway analysis were accessed through the Genetic Association Information Network (GAIN). In brief, the MDD GWA data included 1,738 cases and 1,802 controls of Western European ancestry with 435,291 SNPs (single nucleotide polymorphisms); the BPD GWA data included 1,001 cases and 1,033 controls of European Americans with approximately 724,067 SNPs after data cleaning. More detailed information about the GAIN GWA datasets is available elsewhere [17, 18]. In total, there were 16,924 mapped genes in the GAIN-MDD dataset and 15,213 mapped genes in the GAIN-BPD dataset.

#### Pathway analysis

We conducted pathway analyses using both competitive and self-contained methods. Competitive tests compare the statistics of certain genes in a given pathway with genes not in the pathway, while self-contained methods only compare specific genes in an interested pathway with non-associated background genome. Thus, we applied both the GSEA (Gene set enrichment analysis) and SumStat (sum statistic) methods for pathway analysis. Detailed information on these two statistics can be found elsewhere [19, 20]. In brief, the GSEA method calculates the enrichment score (ES) for the genes in pathways and compares the scores of other genes outside the pathway to evaluate the associations, while SumStat sums over all statistics in a gene set. The results were similar using the two methods, and thus, we report the results using the SumStat method in result Table.

To further consider the information gathered from PPI, we implemented a weighting scheme to give I-Genes twice the weight of the remaining genes in a given pathway. The significance of each examined pathway was calculated by 15,000 times permutation. The analysis flow chart for pathway analysis is shown in S2 Fig. To avoid the bias that larger genes may have smaller p-values by chance, we adopted the method of FOSCO (First Order Statistic Correction) to calculate gene-size corrected p-values for all genes [21, 22]. For multiple testing correction for the 61 examined pathways, we used the Benjamini-Hochberg method to adjust for p-values [23]. A p_adj_-value less than 0.05 was considered significant. All the pathway analyses were performed using R (2.14.2).

## Results

### Identity rate and sequence alignments in *BDNF* gene

The DNA length of the BDNF gene varies widely across the examined vertebrates (Table 1), ranging from 770 base pairs in the finch to ~70,000 base pairs in primates. This variation may be partly due to DNA sequence repeats, as a higher repeat percentage was observed in longer DNA lengths. However, the DNA sequence of CDS and the amino acid sequence are directly related to protein coding, and non-coding sequences are removed during certain biological processes. Consequently, the variations in the DNA length of CDS and the amino acid length are relatively small. In molecular evolution, nucleotides are considered to be more informative, while amino acids are closer to the real biological function [24]. The higher proportion of repeats observed in nucleotide sequences was consistent with the complexity of the organisms we examined. On the other hand, non-coding region variations are suggested to play roles in advanced regulatory functions to affect the expression of coding genes [25]. Thus, variations in the non-coding region may also have impacts on BDNF availability.

**Table 1:** *BDNF* genes in 12 species.

Species	Gene ID	DNA		CDS			Amino acid	
		Length (bp)	Repeat (%)	Length (bp)	Identity (%)	Type	Length (aa)	Identity (%)
Human	627^a^	67166	32.03	768		NM_170731.4	255	
Chimpanzees	503511^a^	---	---	744	99.46	NM_001012441.1	247	100
Macaque	ENSMMUG00000008634^b^	69759	32.49	4058	99.19	ENSMMUT00000012075	247	100
Dog	403461^a^	1130	2.30	744	93.68	NM_001002975.1	247	98.38
Pig	397495^a^	34370	18.74	759	91.05	NM_214259.1	252	96.83
Cattle	617701^a^	1058	0	753	91.1	NM_001046607.1	250	96
Mouse	12064^a^	52344	18.82	774	91.88	NM_007540.4	257	96.89
Rat	24225^a^	50535	13.16	750	91.62	NM_012513.3	249	96.79
Finch	751584^a^	770	---	741	84.97	NM_001048255.1	246	90.28
Turkey	ENSMGAG00000015359^b^	---	---	840	86.21	ENSMGAT00000017275	246	92.31
Chicken	396186^a^	840	0	741	85.7	NM_001031616.1	246	91.5
Zebrafish	58118^a^	7736	4.07	813	---	NM_131595.2	270	70.04

^a^ NCBI Entrez Gene database;

^b^ Ensembl database; CDS: coding DNA sequence

Chimpanzee: Pan troglodytes; Macaque: Macaca mulatta; Pig: Sus scrofa; Dog: Canis lupus familiaris; Cattle: Bos Taurus; Mouse: Mus musculus; Rat: Rattus norvegicus; Finch: Taeniopygia guttata; Turkey: Meleagris gallopavo; Chicken: Gallus gallus; Fish: Danio rerio

**Fig 1:**
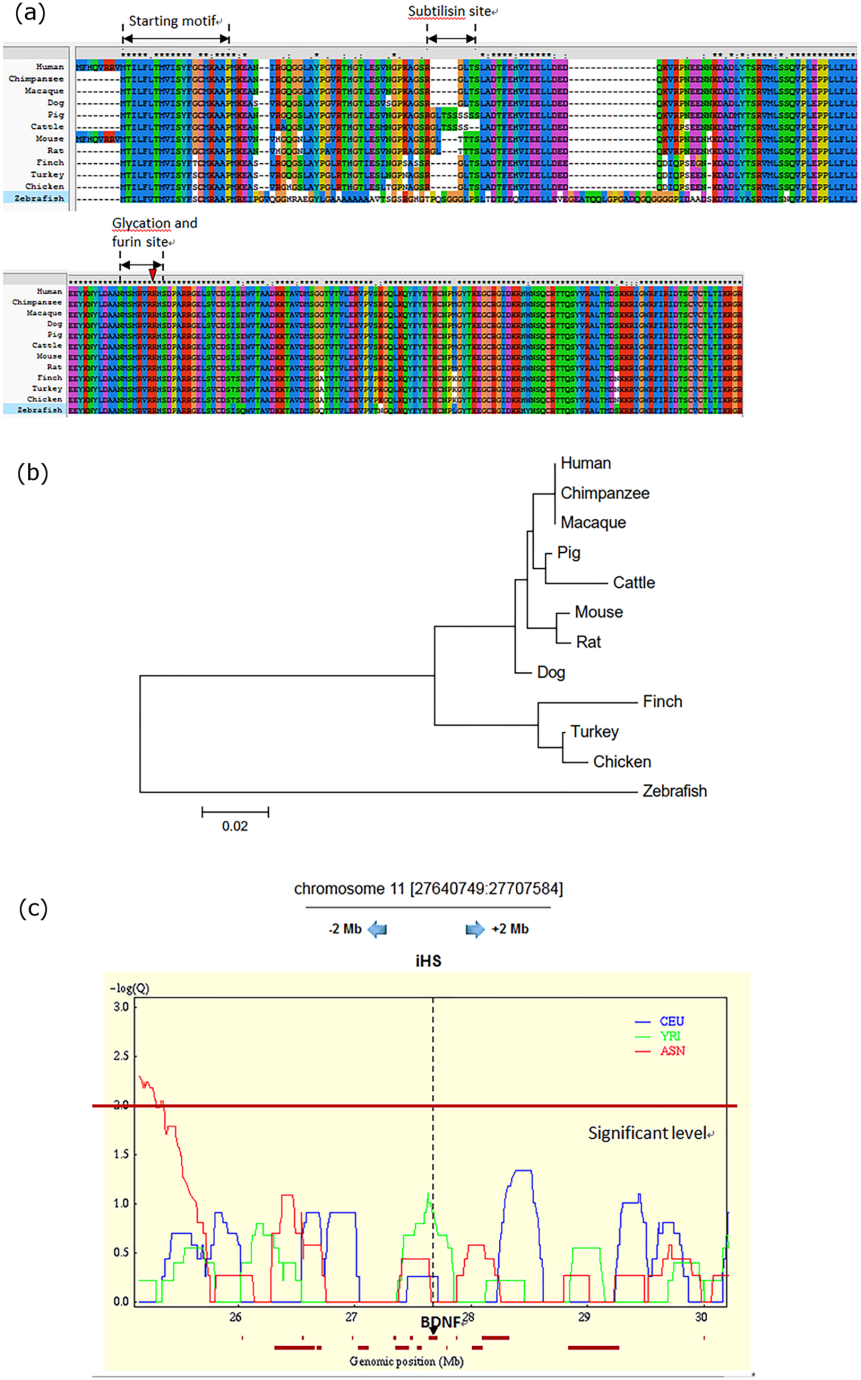
The multiple alignment (a) and Evolutionary tree of BDNF (b) in 12 species, and the positive selection in BDNF(c). (a) The multiple alignment of BDNF sequence in 12 species by ClustalX. Arrowhead indicates the proteolytic site for specific enzymes to produce mature BDNF(*: Fully conserved) (b) Evolutionary tree of BDNF in 12 species produced by neighbor-joining methods in MEGA 5 (c) Plot showed the positive selection in BDNF by iHS score in Happlotter (Significant level: iHS ≧2; CEU: Utah residents with northern and western European ancestry from the CEPH collection; YRI: Yoruban in Ibadan, Nigeria; ASN: East Asians).

Using the human sequence as the reference, the CDS sequence identity rate is high across species. For example, the identity rate is 85.7% between humans and chickens. The identity rate in the amino acid sequence between humans and other species is even higher, with an identity above 90% for chickens and a low in zebrafish of 70%. Except for zebrafish, the *BDNF* gene is highly conserved among the vertebrates examined. The high identity rate in amino acid sequences across species suggested that the functions of *BDNF* originated early in evolutionary history and are not specific to mammals with more complicated and sophisticated emotional expression. Fig 1b displays the evolutionary tree of the examined species based on BDNF protein sequences. Our results demonstrate a clear separation between mammals and non-mammals, as well as between primates and non-primates, which is consistent with the expectation for species relationships.

The results of multiple alignments are shown in Fig 1a. The specific splicing site for producing mature BDNF was fully conserved across all examined species, and the sequence of mature BDNF was almost completely conserved in mammals. Similarly, several domains before the splicing site were also conserved in mammals, except the subtilisin site, which is consistent with findings in Tettamanti, Cattaneo (12]. The region after the specific splicing site is defined as a nerve growth factor domain, which has been found to be conserved in other neurotrophin family members [26]. In other words, multiple alignments revealed much variation in the pro-BDNF region, where the most studied polymorphism Val66Met (rs6265) is located [27]. This finding indicates the importance of studying pro-BDNF rather than m-BDNF alone. Considering the biological process from the gene level to different protein products might be necessary in studying BDNF and its phenotypes, including mood disorders.

### Testing for recent positive selection

Human possess a higher level of emotional expressions and complex cognitional function; thus, we examined adaptive selection across species as well as several human populations. The dN/dS ratios in pairwise comparisons across species were all below one (S1 Table.), indicating that the *BDNF* gene did not experience strong natural selection in these species. Fig 1c showed the positive selection of *BDNF* variants in the CEU, YRI and ASN populations. In the *BDNF* gene region, the selection force was highest in the YRI population, with the highest differential linkage disequilibrium. However, the magnitude of positive selection in all three populations was non-significant (|iHS|<2). Moreover, we applied another program, dbPSHP [28], which utilized HapMap phase III data to examine selective signals in 11 populations. No significant positive selection was found for *BDNF* region in all examined populations.

The findings of non-significant natural selection in the evolutionary history across species and different human populations were consistent with results reported using the Selectome database [29]. The absence of significant selection for the *BDNF* gene across species and human populations suggested that the polymorphisms in this gene are unlikely to have dramatic deleterious impacts on species fitness and survival. Interestingly, a recent study reported high genetic diversity for the Val66Met polymorphism, of which the derived Met allele has frequency ranging from 0–72% in 58 global populations [30], suggesting the possibility for local selection. The same study then detected selection signal in a common 45% frequency haplotype carrying the Val66 allele in 12 European-derived CEPH pedigrees. Nevertheless, whether similar selection signals could be detected in other population data is unclear. The fact that we did not find positive selection among all examined populations in HapMap phase II and III data could be due to several reasons, such as marker density in the *BDNF* gene regions in the database and different analysis algorithms. However, from the evolutionary genetic point of view, mutations in the *BDNF* gene may still influence adaptions to environmental changes, especially for mental disorders. A polygenic mutation-selection balance theory (i.e., mutations in a large number of genes with accumulated influence on human behaviors) has been suggested to explain the development of mood disorders in human [31], further indicating the necessity of considering other genes and genes that interact with *BDNF*.

### Systematic literature review of genomic studies on the relationship between *BDNF* and mood disorders

A decade ago, the neurotrophin hypothesis was proposed for the etiology of mood disorders, and BDNF is among the most studied neurotrophic factors [32]. A variety of studies have been conducted to investigate the relationships between BDNF and mood disorders. In this section, we included evidence from studies measuring BDNF serum levels, gene expression studies, and linkage and genetic association studies of *BDNF*. The results of this literature review of the genomic study designs of the *BDNF* gene and serum/plasma levels are listed in Table 2. For gene expression studies, no significantly different *BDNF* mRNA levels in peripheral blood were found between BPD patients and controls [33]. We also analyzed empirical gene expression data in postmortem brains of 12 studies in MDD and BPD from SMRI database. A consensus fold change could be obtained based on a weighted combination across different studies for each gene in the SMRI database. Results showed that the fold change of BDNF was small and p-values were non-significant for BPD (fold change = -1.03, p-value = 0.10) and MDD (fold change = 1.03, p value = 0.35). We found that *BDNF* mRNA level was neither associated with mood disorders nor exhibited substantial fold change between mood disorder patients and healthy controls.

**Table 2:** Systematic reviews of genetic studies of *BDNF* in review or meta- type articles (ordered by publication year).

Disease	Study design	Author	Description of study	Results direction	Results description
**BPD**	Gene expression study	Munkholm K et al. (2012)[33]	Review article.	Negative	There was no significant BDNF mRNA level difference between patients and controls in three studies.
	Linkage study	Craddock N et al. (2005)[68]	Review article.	Negative	None of the region reviewed in the article included *BDNF* gene.
		Hayden EP et al. (2006)[34]	Review article.	Positive	Multiple susceptibility loci with 2p, 4p, 4q, 6q, 8q, 11p, 12q, 13q, 16p, 16q, 18p, 18q, 21q, 22q, and Xq.
		Serretti A & Mandelli L (2008)[69]	Review article.	Negative	Regions associated with BP didn’t include loci 11p13, which was the region that *BDNF* located.
		Craddock N et al. (2009)[70]	Review article.	Negative	Reviews of previous meta-analysis showed different susceptible regions, and none of them included loci of *BDNF*.
	Association study	Kanazawa T et al. (2007)[36]	Meta-analysis of 11 studies with 3,143 cases and 6,347 controls.	Negative	The association between BDNF rs6265 and BPD was not significant.
		Seifuddin F et al. (2012)[37]	Meta-analysis of 12 studies with 3,897 cases and 6,807 controls.	Positive	The top polymorphism rs6265 in BDNF was showed nominally significant (p-value<0.05), but it was not significant after correction.
	GWA study	Scott LJ et al. (2009)[38]	Meta-analysis with 3,683 cases and 14,507 controls.	Negative	No SNP reached the genome-wide significance (P<5x10^-8^) in 3-study meta-analysis.
	Serum /Plasma BDNF level	Lin PY (2009)[45]	Meta-analysis of 15 studies with 122 mania cases, 67 depressive cases and 273 controls.	Positive	The BDNF levels in blood were significant lower in manic and depressed state of BPD cases than controls.
		Fernandes BS et al. (2011)[47]	Meta-analysis of 13 studies on both serum and plasma levels with 122 mania cases and 128 controls, and 107 depressive cases and 118 controls.	Positive	BDNF levels were significantly lower in manic, depressive BPD patients than in controls.
**MDD**	Linkage study	Levinson DF (2006)[71]	Review article.	Negative	Linkage findings with more than one study support didn’t include the *BDNF* gene.
		Lohoff FW (2010)[72]	Review article.	Negative	The author reviewed five large sample studies, but none of them observed a significant linkage region of *BDNF*.
	Association study	Verhagen M et al. (2010)[35]	Meta-analysis of 14 studies with 2,812 cases and 10,843 controls.	Positive	Weak association between BDNF rs6265 polymorphism and depression can be observed in men.
	GWA study	Wray NR et al. (2012)[41]	Meta-analysis of 3 studies with 5,763 cases and 6,901 controls.	Negative	Rs6265 did not reach significant level in the meta-analysis of three largest MDD GWAS.
		MDD Working Group of the PGC^a^ (2012)[42]	Mega-analysis with 9,240 cases and 9,519 controls in discovery phase and 6,783 and 50,695 controls in replication phase.	Negative	No SNP reached the genome-wide significance in discovery and replication phase.
	Serum /Plasma BDNF level	Brunoni AR et al. (2008)[46]	Meta-analysis of 20 studies with 1,504 cases.	Positive	Serum and plasma BDNF levels in blood increased after treatments, and negatively correlated with severity.
		Sen S et al. (2008)[43]	Meta-analysis of 11 studies with 366 cases and 382 controls.	Positive	Serum BDNF levels in MDD patients were significant lower than in controls.
		Bocchio-Chiavetto L et al. (2010)[44]	Meta-analysis in total of 15 studies with 489 cases and 483 controls on serum studies, and 161 cases and 211 controls on plasma studies.	Positive	Both BDNF serum and plasma level were significantly lower in MDD patients than in controls.
**Mood disorders**	Linkage study	Craddock N et al. (2005)[68]	Review article.	Negative	Studies in BP indicated strongest susceptibility loci on 13q and 22q; 12q22-23 and 2q in unipolar depression.
	GWA study	McMahon FJ et al. (2010)[39]	Meta-analysis of 5 studies with 6,683 cases and 9,068 controls.	Negative	The p-value of rs6265 in the meta-analysis is 0.7516.
		Liu Y et al. (2011)[40]	Meta-analysis of 2 studies with 6,082 cases and 7,970 controls.	Negative	Rs6265 was not in the list of SNP with P<10^-5^.

^a^ Major depressive disorder working group of the psychiatric GWAS consortium

Linkage studies are often adopted to examine susceptible genetic regions and loci for the trait of interest. The *BDNF* gene is located on chromosome 11p. In seven review articles of linkage studies for MDD and BPD, only one study on BPD reported a suggestive linkage region at 11p (with LOD score of 2, near marker D11S1923) [34]. However, this region is distant from the *BDNF* gene (greater than 130 Mbp). Thus, the results from linkage studies provided negative evidence for mutations of the *BDNF* gene to have any direct and substantial effect on the familial inheritance of mood disorders, which may be due to the complex genetic etiology underlying MDD and BPD. The effect of the *BDNF* gene on mood disorder is too weak to be detected by family-based linkage study design.

For genetic association studies, two designs are commonly adopted: the candidate genes approach and the GWA approach. In the *BDNF* gene, the most tested genetic polymorphism is the Val66Met variant (rs6265). A few meta-analysis studies have been conducted for MDD and BPD, and the results are summarized in Table 2. Verhagen et al., (2010) selected 14 individual studies examining the Val66Met marker in 2,812 MDD cases and 10,843 healthy controls [35]. While the association between Val66Met and MDD in total samples was non-significant, gender stratification analysis showed an association in males only (OR = 1.67, P<0.01 in genotypic model). For BPD, an early meta-analysis with 11 studies did not find a significant effect of Val66Met [36]. Another study using 3,897 BPD cases and 6,807 controls found nominal significance for the marker Val66Met, but the association became non-significant after correcting for multiple testing [37].

Large scale GWA studies are expected to have a better chance of finding underlying susceptible genetic variants for mood disorders, especially meta- or mega-analysis of GWA studies that include even larger sample sizes with millions of markers distributed over the genome. Several GWA meta-analyses with thousands of individuals have been conducted for MDD and BPD, however, none of these studies reported suggestive signals for the association of the Val66Met marker or other genetic variants of *BDNF* with mood disorders [38–42]. These somewhat inconsistent and majorly negative findings from genetic association studies are unlikely to result from the low power of individual studies, as a trend of no association was found in most of the meta-analysis studies. Petryshen et al. (2010) has observed considerable global diversity of BDNF SNPs. This genetic diversity somewhat decreases the sample size and reduces the analysis power in meta-analysis but is also unlikely to have caused the inconsistent results.

Some studies have examined the relationship between BDNF protein level and neuropsychiatric disorders in the hope of identifying useful peripheral biomarkers for predicting the risk of mood disorders. While most of the genomic studies (such as gene expression, linkage, or association studies) showed negative or inconsistent results, protein level studies seemed to demonstrate more consistent and positive findings. We systematically reviewed articles and meta-analysis studies for MDD and BPD considering both plasma and serum BDNF expression levels. For MDD, a significant association (P<6x10^-8^) between serum BDNF and depression status was also reported in Sen et al., (2008) [43]. In a meta-analysis with a larger sample size, findings in Bocchio-Chiavetto et al., (2010) supported a low serum BDNF level in MDD patients, and a similar pattern was found in the plasma BDNF level with 161 MDD patients and 211 controls [44]. For BPD, Lin et al., (2009) included 15 studies in a meta-analysis that showed significantly lower BDNF levels in blood in BPD patients than in controls (P = 1x10^-4^) [45].

In addition to the comparison between the blood level of BDNF in mood disorder patients and healthy controls, several studies further investigate the effect of different disorder statuses. After medication treatment, significantly increased BDNF levels were found in MDD patients [43, 46]. For BPD, two studies compared BDNF level in different mood episodic statuses with controls. Their results found that BDNF levels were significantly lower in patients in manic and depressed acute states but not in euthymic state [45, 47]. Moreover, the BDNF level increased significantly after treatment in patients with acute manic episodes [45], further supporting the direct association of BDNF protein level with mood episodic status. In contrast to genomic studies that investigate the relationship between gene and mood disorders, the protein level of BDNF seemed to be closer to behavioral expression and may have a more direct effect on mood disturbances. Based on reviewing the evidence in the literature, we considered that the more consistent findings for the involvement of BDNF in mood disorders come from protein level studies, which suggest roles for BDNF in the mechanisms of mood disorders.

### Data integration

Although *BDNF* has been widely tested for mood disorders, the evidence was not consistent across different study designs and individual studies. A data integration strategy is necessary to evaluate the role of *BDNF* in mood disorders. We previously developed a data integration pipeline to combine multiple sources of genomic information (e.g., association, linkage, gene expression, regulatory pathway) to prioritize candidate loci for MDD [7]. With a modified algorithm, we found that BDNF was listed as the top depression candidate gene among the 151 identified DEPgenes (candidate gene list for MDD in Kao et al., (2011) [7]. Similarly, *BDNF* was identified in the top candidate gene list for BPD [22, 48]. Therefore, it is preferable to consider an integrative framework to accommodate a large amount of genomic data from different sources rather than focusing on one specific study design to weight the influences of *BDNF* in mood disorders.

### Pathway analyses for BDNF and its interacting molecules

It is possible that BDNF interacts with other molecules in specific biological pathways to exert its effect on the trait of interest [49]. We further performed pathway analyses to examine BDNF-related pathways with MDD and BPD separately. Using GAIN GWA datasets for MDD and BPD with pathways collected in MSigDB, we reported significant pathways for mood disorders involved with BDNF (BDNF-pw) and its interacting molecules (I-Genes) in Table 3. In total, we identified 29 BDNF-pw and 34 IGene-pw. Two pathways overlapped between the IGene-pw and BDNF-pw sets: the MAPK signaling pathway and the neurotrophin signaling pathway (S2 Table.). The median gene number was 103 (ranging from 13 to 373) in the 29 BDNF-pw and 87.5 (ranging from 16 to 377) in the 34 IGene-pw.

**Table 3:** The significant pathways after gene size adjustment in 34 IGene-pw and 29 BDNF-pw of pathway analysis by using GAIN-MDD or GAIN-BPD GWAS data and SumStat methods.

Pathway name	# of games^a^	% of I-Genes	MDD		BPD	
			Non-weighting	Weighting	Non-weighting	Weighting
**34 IGene-pw**						
Source:KEGG						
Amyotrophic lateral sclerosis als	53	39.62	0.1161	0.0947	0.0847	**0.0468**
Calcium signaling pathway	178	17.98	0.0631	**0.0286**	**0.0018** *	**0.0009** *
Focal adhesion	201	12.44	0.4775	0.4812	**0.0023** *	**0.0025** *
Long term potentiation	70	31.43	0.2641	0.1156	**0.0061** *	**0.0064** *
MAPK signaling pathway	267	14.23	0.4849	0.4288	**0.0255**	**0.0303**
Neuroactive ligand receptor interaction	272	16.91	0.4573	0.4085	**0.0110** *	**0.0053** *
Pathways in cancer	328	10.06	0.4865	0.5610	**0.0025** *	**0.0025** *
Source:Reactome						
Activation of NMDA receptor upon glutamate binding and postsynaptic events	36	44.44	0.1094	0.0807	**0.0404**	**0.0425**
Neuroransmitter receptor binding and downstream transmission in the postsynaptic cell	84	20.24	0.1867	0.1104	**0.0222**	**0.0207**
Post NMDA receptor activation events	32	40.63	0.1284	0.0937	**0.0395**	**0.0379**
Signalling by NGF	215	17.21	0.5898	0.5555	**0.0129** *	**0.0253**
Transmission across chemical synapses	130	20.77	0.0779	0.0755	**0.0057** *	**0.0159**
Trka signaling from the plasma membrane	103	17.48	0.6334	0.5771	**0.0189**	**0.0325**
Source:GO term						
G protein coupled receptor protein signaling pathway	326	11.04	0.8365	0.7693	**0.0099** *	**0.0103** *
Glutamate receptor activity	20	70.00	**0.0237**	**0.0311**	**0.0220**	**0.0293**
Glutamate signaling pathway	17	52.94	0.1629	0.2065	**0.0401**	**0.0586**
Neurological system process	377	9.81	0.1955	0.1073	**0.0001** *	**0.0003** *
Synaptic transmission	172	18.60	0.0935	**0.0429**	**0.0007** *	**0.0007** *
Transmission of nerve impulse	187	17.11	0.1297	0.0585	**0.0014** *	**0.0011** *
**29 BDNF-pw**						
Source:KEGG						
MAPK signaling pathway	267	14.23	0.4925	0.4284	**0.0183**	**0.0255**
Source:GO term						
Receptor binding	373	6.17	0.9845	0.9757	**0.0031** *	**0.0035**
Source:Curated gene sets						
Browne HCMV infection 10hr dn	57	3.51	0.1384	0.1548	**0.0099**	**0.0123**
Charafe breast cancer basal vs mesenchymal dn	51	1.96	0.0519	0.0550	**0.0150**	**0.0201**
Dang regulated by MYC dn	243	4.94	0.8826	0.8694	**0.0395**	**0.0503**
Han SATB1 targets dn	331	1.81	0.9534	0.9441	**0.0053**	**0.0065**
Hellebrekers silenced during tumor angiogenesis	56	5.36	0.0914	0.1043	**0.0288**	**0.0232**
Kaab heart atrium vs ventricle dn	267	1.12	0.1065	0.0965	**0.0001** *	**0.0002** *
Takeda targets of NUP98 HOXA9 fusion 8d up	157	3.18	0.3365	0.3824	**0.0479**	**0.0331**

^a^ The total number of genes in each pathway annotated by MsigDB.

Bold: p-value<0.05

* Pathways which are still significant after multiple testing adjustment

Table 3 shows that there were 19 nominally significant IGene-pw and 9 significant BDNF-pw that were associated with mood disorders. However, after correction of the multiple testing issue, none of the pathways were significant for MDD, while 11 IGene-pw and 2 BDNF-pw were still significant in BPD. Considering the high heritability in BPD, more loci might be involved in corresponding with much complex pathogenesis for BPD. This result implied the importance of considering interacting proteins to explore the roles of *BDNF* and the underlying mechanism of BPD. For both MDD and BPD, the results were very similar regardless of adding a weighting scheme to I-Genes or not. Adding extra weight to the genes that interact with *BDNF* in specified pathways seemed to be irrelevant and did not exert substantial influence on the pathway analysis results.

Comparing significant pathways of IGene-pw and BDNF-pw, we found that the results were quite different. The most significant IGene-pw were the calcium signaling pathway, neurological system process, and synaptic transmission for BPD (P<0.001), which were all related to nervous system functions. In contrast, the significant BDNF-pw were related to receptor binding, gene regulations of certain cells or organisms that were involved in cancer or physical health problems. Several previous studies have shown that cancer mortality rates were higher in mood disorder patients than in the general population [50]. The BDNF-pw results suggest a research avenue to study the link between mood disorders and cancers and to explore the common roles of *BDNF* in the two disease categories. Interestingly, the MAPK signaling pathway that included both the *BDNF* gene and more than 10 percent IGenes showed association with BPD under both the BDNF-pw and IGene-pw categories. The MAPK pathway is activated by *BDNF* through binding to the TrkB receptor, which further emphasizes the involvement of BDNF in mood disorders.

Some of the significant pathways that were identified in MDD and/or BPD in our studies were also reported by previous studies for mood disorders, such as calcium signaling pathway and glutamate receptor activity. Hasbi et al., (2009) reported that calcium mobilization to the plasma membrane was induced by the activation of the dopamine D1-D2 receptor heteromer, and the mobilization triggered the activation of CaMKIIα in the nucleus that enhanced BDNF expression [51]. BDNF also plays a role in the modification of glutamate synapses through regulating glutamate secretion presynaptically and changing the phosphorylation of glutamate receptor through postsynaptic actions [52]. A more detailed discussion of the roles of glutamate in emotion, cognition and depression can be found in a recent review [53]. To summarize, we identified a few significant biological pathways for mood disorders that involve BDNF and BDNF-interacting molecules. Studying these enriched pathways in future work might facilitate our understanding of the underlying etiology of mood disorders and how BDNF and its interacting molecules function to influence the risk of mood disorders.

### The roles of pro-BDNF and m-BDNF in mood disorders

Neurotrophins are first produced and released in a precursor form and then proteolytically cleaved by several enzymes to create a mature form [27]. Like other neurotrophins, pro-BDNF is cleaved to m-BDNF within the endoplasmic reticulum by furin or in regulated secretory vesicles by proconvertase enzymes. When pro-BDNF is released to the extracellular environment, it is then cleaved by plasmin [54]. The activities of these cleavage enzymes critically decide the downstream actions, triggering cell survival by m-BDNF or apoptosis by pro-BDNF. Due to the opposite biological functions of pro-BDNF and m-BDNF in the neuronal system, it is important to discuss the roles of these two proteins in relation to mood disorders. In addition, the factors and contributors that lead to the cleavage of pro-BDNF to m-BDNF are also key points in understanding the mechanism of mood disorders. For example, it has been postulated that BDNF affects mood disorders through protein p11 by activating tissue plasminogen activator (tPA)/plasminogen activity, cutting pro-BDNF to increase the production of m-BDNF and leading to an antidepressant effect [55]. Therefore, enzymes that influence (tPA)/plasminogen activity are other potential targets to investigate for mood disorders.

On the other hand, the level of BDNF is found to be influenced by adverse environmental stimuli and stress, which is often linked with emotional, physiological and psychological reactions. Acute and chronic stresses are reported to increase the risk of developing mood disorders [32, 56], and several studies had showed the negative environmental stimuli effect on the relationship between BDNF and depression [57, 58]. A negative relationship between stress and BDNF blood level is found in depression patients [59]. Moreover, animal models are often applied to investigate the effects of stress on the level of BDNF. Using a zebrafish model (its brain organization is similar to higher vertebrates) to directly examine the effect of stress on neurotrophins, Tognoli et al., (2012) reported alterations of BDNF levels after acute stress, where the pro-BDNF level was increased and the m-BDNF level was decreased [60]. The authors further suggested that the pro-BDNF/total-BDNF ratio could accurately predict stress response. Several other studies have conducted experiments in rats. In one recent study, prenatal stress was employed in different rat strains, and changes in the protein levels of pro-BDNF and m-BDNF, as well as cleavage-related protease and BDNF downstream signaling proteins, were found after prenatal stress [61]. Notably, these changes depend on strains, revealing the critical role of genetic background. Another experiment used a chronic prenatal restraint stress model in rats to explore the effect of prenatal stress on hippocampal synaptic plasticity in the offspring [62]. Decreased protease tPA expression was found, which decreased the proteolytic cleavage from pro-BDNF to m-BDNF, leading to increased levels of pro-BDNF and decreased levels of m-BDNF in rats, thereby mediating the effect of prenatal stress on long-term potentiation and long-term depression in the hippocampus. These aforementioned study findings indicated the influence of stress on individuals by altering the levels of pro-BDNF and m-BDNF directly or indirectly through the cleavage enzymes. In addition, this evidence also supports the involvement of both pro-BDNF and m-BDNF in the etiological mechanisms of mood disorders. Thus, how individuals respond to acute or chronic stress may involve the regulation of BDNF-related pathways. Dysfunction of the BDNF-related pathways might be one of the key points leading to mood disorders in response to environmental stimuli.

Other observations regarding anti-depression treatment provide additional supporting evidence for the roles of BDNF in mood disorders. Segawa et al., (2013) examined the effects of electroconvulsive seizures (ECS) on the expression of BDNF and other protease enzymes in the hippocampus of rats [63]. The authors found that ECS increased the levels of pro-BDNF and extracellular protease (t-PA) simultaneously, as well as enhancing the pro-BDNF processing and thereby increasing the level of m-BDNF to exert its antidepressant effect. This process may partly explain the clinical effectiveness of ECS in treating depression compared to antidepressants. Despite the opposite functions of pro-BDNF and m-BDNF, they are rarely distinguished in previous studies examining the serum/plasma BDNF level. It was not until recently that one study tested pro-BDNF and m-BDNF levels in human saliva, and the authors also reported that the m-BDNF level was altered by the Val66Met polymorphism [64]. In 2012, Yoshida et al. used a newly available human pro-BDNF and m-BDNF ELISA kit to examine the BDNF serum level in MDD patients [65]. They found that m-BDNF, but not pro-BDNF, is lower in MDD patients than in healthy controls. However, the sensitivity of measuring pro-BDNF is much lower than the sensitivity of measuring m-BDNF in Yoshida et al.’s study. To overcome this measurement technique issue would assist in studying the mechanisms of BDNF-related molecules and pathways in mood disorders.

The current study had certain limitations. Because most of our analyses relied on data from publicly available and published literature reports, the accuracy of these results is influenced by the completeness of the databases and potential publication bias in the literature. This situation exists in most similar research settings, and we elected to use multiple databases to reduce the potential problems. We selected a so-called integrated database to reduce information bias and restricted our literature review to meta-analysis and review studies. In addition, we collected data from different data sources as completely as possible and developed a weighting system in the hope of obtaining more robust findings.

## Conclusions and Future Directions

From a biological perspective, BDNF proteins participate in neuron development and stress responses, which are considered to play important roles in relation to neuropsychiatric disorders. Many previous studies examined the relationship between BDNF and mood disorders using a variety of study designs and analytical methods. We have systematically reviewed evidence from genomic studies and BDNF protein level studies, including pro-BDNF and m-BDNF. In addition, we performed sequence alignment among different species and tested positive selection to explore the evolutionary history of *BDNF*. Genes that interact with *BDNF* are evaluated at the pathway level and analyzed using two GWAS datasets of BPD and MDD.

To summarize, we observed a general pattern that most linkage and GWA association studies provided negative findings for *BDNF* with mood disorders, while studies examining serum/plasma levels of BDNF provided more consistent positive findings for its involvement in mood disorders. The lack of evidence from genetic studies may not negate the roles of *BDNF* in mood disorders. Most of the risk variants in GWA studies often explain very small proportion of heritability in complex disorders, and the negative findings may result from small main effect of *BDNF* [66]. In addition, multiple-gene interactions are not usually considered in most genetic studies. The significant findings in our pathway analysis (especially when we considered the I-genes) suggest that the association of *BDNF* with mood disorders may not be through the main effect. *BDNF* likely acts as a modifier gene or jointly with other genes in the same pathway to contribute to the risk of mood disorders. One example is a study that reported a significant interaction effect between *BDNF* Val66Met and *DRD3* Ser9Gly genotypes, influencing bipolar disorder [67]. Because of the limitations in each type of study design, strategies that are designed to incorporate multiple lines of evidence are appreciated for the study of the underlying biological functions of targeted genes and molecules for the disorder of interest.

Stress is considered an important environmental stimulus that influences the risk of mood disorders by altering the expression of BDNF. Previous studies have suggested different roles for pro-BDNF and m-BDNF and how these proteins operate in response to stress. Examining the changes in pro-BDNF and m-BDNF blood levels and the pro-BDNF/m-BDNF ratio may be useful and important for the early detection of mood disorders. Moreover, the expression of the proteases that cut pro-BDNF to produce m-BDNF and of the proteins that interact with BDNF may also be important indicators for the risk of developing mood disorders, and further study is warranted to investigate their roles in the underlying mechanisms of mood disorders.

## Supporting Information

S1 Fig The network of proteins interact with BDNF which is based on the evidence score more than 0.9 produced by STRING. (DOCX)

S2 Fig The flow chart of pathway analysis in 34 IGene-pw. **A**. Genes interacts with *BDNF* by STRING. (I-Genes, #:363) **B**. Map I-Genes to the pathways in MsigDB, select the pathways which overlap rate is more than 10%, and exclude pathways with extreme gene numbers. (#:34) **C**. All the genes in 34 pathways were included for analysis. (#:1,818) **D**. Give weights for genes with evidence score provided by STRING. **E**. Use GSEA and SumStat as statistics.
(DOCX)

S1 Table Pairwise dN/dS ratio in the *BDNF* gene across species. Significant level: dN/dS≧1.
(DOCX)

S2 Table The list of pathways that were recruited for pathway analysis in this study. ^a^ The total number of genes in each pathway annotated by MsigDB. ^b^ Pathway was selected since it was related to BDNF. ^c^ Pathway was selected since it include more than 10 percent I-Genes. ^d^ Pathway was significant in at least one statistical way.—: Did not include in pathway analysis since the extreme pathway gene numbers.
(DOCX)
