# Supplementary material for: Explore the Features of Brain-Derived Neurotrophic Factor in Mood Disorders
Source: PLoS One. 2015 Jun 19;10(6):e0128605. doi: 10.1371/journal.pone.0128605 (PMC4474832; doi:10.1371/journal.pone.0128605)

**Figure S1. The network of proteins interact with BDNF which is based on the evidence score more than 0.9 produced by STRING.**


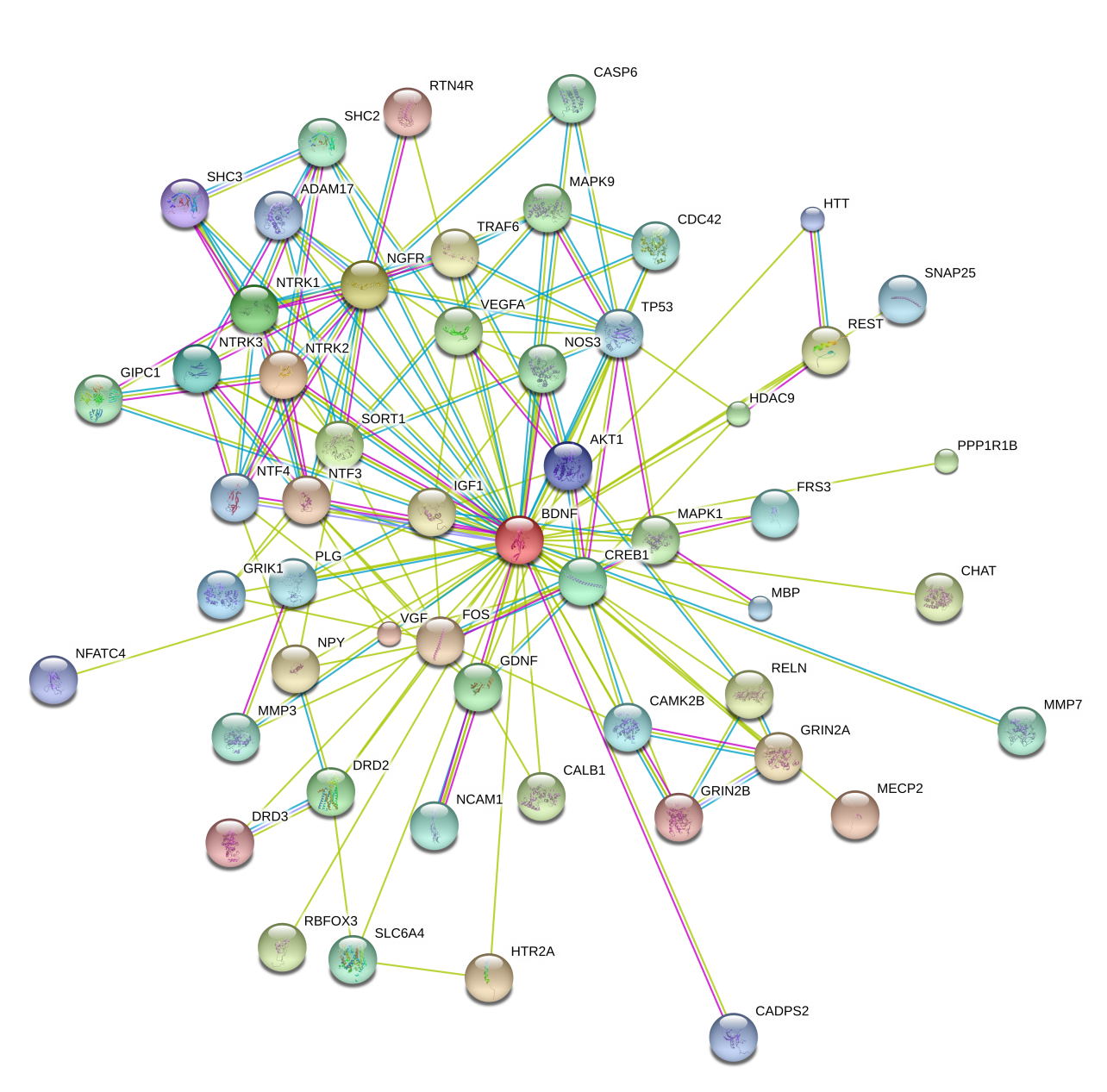

Supplement: S1 Fig — (DOCX) [file pone.0128605.s001.docx]
