# Supplementary material for: Explore the Features of Brain-Derived Neurotrophic Factor in Mood Disorders
Source: PLoS One. 2015 Jun 19;10(6):e0128605. doi: 10.1371/journal.pone.0128605 (PMC4474832; doi:10.1371/journal.pone.0128605)
Supplement: S2 Fig — A. Genes interacts with BDNF by STRING. (I-Genes, #:363) B. Map I-Genes to the pathways in MsigDB, select the pathways which overlap rate is more than 10%, and exclude pathways with extreme gene numbers. (#:34) C. All the genes in 34 pathways were included for analysis. (#:1,818) D. Give weights for genes with evidence score provided by STRING. E. Use GSEA and SumStat as statistics. (DOCX) [file pone.0128605.s002.docx]

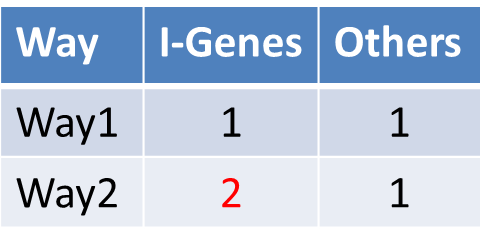

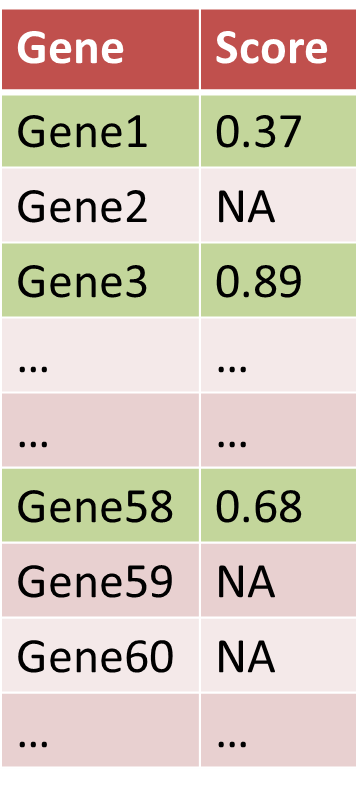

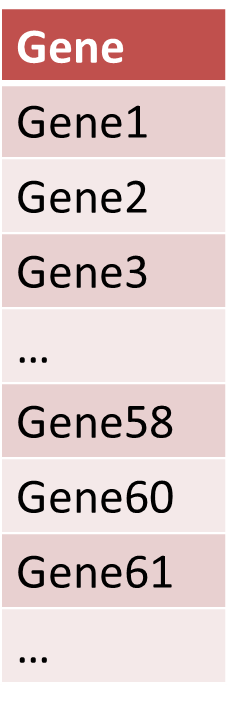

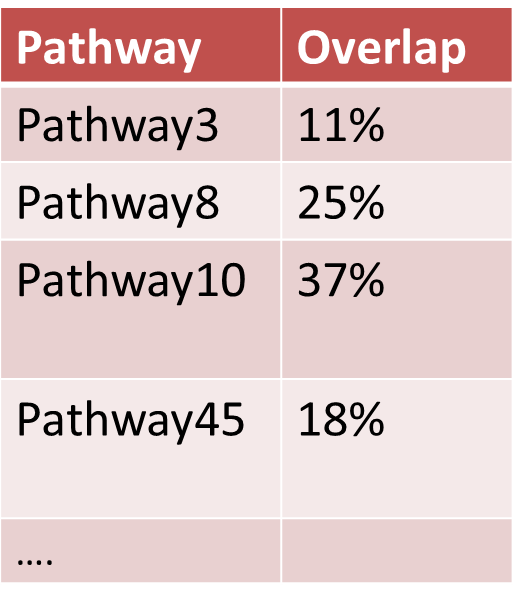

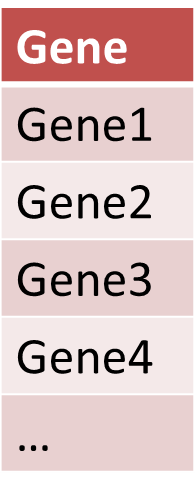


**Figure S2. The flow chart of pathway analysis in 34 IGene-pw.** **A.** Genes interacts with *BDNF* by STRING. (I-Genes, #:363) **B.** Map I-Genes to the pathways in MsigDB, select the pathways which overlap rate is more than 10%, and exclude pathways with extreme gene numbers. (#:34) **C.** All the genes in 34 pathways were included for analysis. (#:1,818) **D.** Give weights for genes with evidence score provided by STRING. **E.** Use GSEA and SumStat as statistics.

**E.**

**A.**

**D.**

**C.**

**B.**

Pathway analysis
