# Supplementary material for: Explore the Features of Brain-Derived Neurotrophic Factor in Mood Disorders
Source: PLoS One. 2015 Jun 19;10(6):e0128605. doi: 10.1371/journal.pone.0128605 (PMC4474832; doi:10.1371/journal.pone.0128605)
Supplement: S1 Table — Significant level: dN/dS≧1. (DOCX) [file pone.0128605.s003.docx]

**Table S1 . Pairwise dN/dS ratio in the *BDNF* gene across species.**

|  | Human | Chimp | Macaque | Dog | Pig | Cattle | Mouse | Rat | Finch | Turkey | Chicken |
| --- | --- | --- | --- | --- | --- | --- | --- | --- | --- | --- | --- |
| Human |  |  |  |  |  |  |  |  |  |  |  |
| Chimp | 0.0010 |  |  |  |  |  |  |  |  |  |  |
| Macaque | 0.0010 | 0.0010 |  |  |  |  |  |  |  |  |  |
| Dog | 0.0291 | 0.0290 | 0.0324 |  |  |  |  |  |  |  |  |
| Pig | 0.0162 | 0.0168 | 0.0184 | 0.0189 |  |  |  |  |  |  |  |
| Cattle | 0.0373 | 0.0379 | 0.0388 | 0.0497 | 0.0326 |  |  |  |  |  |  |
| Mouse | 0.0422 | 0.0436 | 0.0470 | 0.0411 | 0.0295 | 0.0484 |  |  |  |  |  |
| Rat | 0.0456 | 0.0469 | 0.0495 | 0.0461 | 0.0325 | 0.0545 | 0.0460 |  |  |  |  |
| Finch | 0.0692 | 0.0693 | 0.0731 | 0.0668 | 0.0745 | 0.0665 | 0.0646 | 0.0683 |  |  |  |
| Turkey | 0.0543 | 0.0601 | 0.0573 | 0.0440 | 0.0526 | 0.0471 | 0.0506 | 0.0580 | 0.0580 |  |  |
| Chicken | 0.0600 | 0.0637 | 0.0633 | 0.0448 | 0.0583 | 0.0535 | 0.0561 | 0.0608 | 0.0793 | 0.0400 |  |
| Zebrafish | 0.0870 | 0.1059 | 0.1025 | 0.1112 | 0.1182 | 0.1198 | 0.1233 | 0.1291 | 0.1172 | 0.1208 | 0.1107 |

Significant level: dN/dS≧1
