# Supplementary material for: Explore the Features of Brain-Derived Neurotrophic Factor in Mood Disorders
Source: PLoS One. 2015 Jun 19;10(6):e0128605. doi: 10.1371/journal.pone.0128605 (PMC4474832; doi:10.1371/journal.pone.0128605)
Supplement: S2 Table — a The total number of genes in each pathway annotated by MsigDB. b Pathway was selected since it was related to BDNF. c Pathway was selected since it include more than 10 percent I-Genes. d Pathway was significant in at least one statistical way.—: Did not include in pathway analysis since the extreme pathway gene numbers. (DOCX) [file pone.0128605.s004.docx]

**Table S2. The list of pathways that were recruited for pathway analysis in this study.**

| Pathways | # of genes^a^ | % of I-Genes | Select from BDNF^b^ | Select from I-Genes^c^ | Significant in MDD^d^ | Significant in BPD^d^ |
| --- | --- | --- | --- | --- | --- | --- |
| BioCarta |  |  |  |  |  |  |
| ERK5 pathway | 18 | 44.44 | ● |  |  |  |
| NFAT_pathway | 54 | 24.07 |  | ● |  |  |
| KEGG |  |  |  |  |  |  |
| Alzheimers_disease | 169 | 14.20 |  | ● |  |  |
| Amyotrophic lateral sclerosis ALS | 53 | 39.62 |  | ● |  | ● |
| Apoptosis | 88 | 19.32 |  | ● |  |  |
| Calcium signaling pathway | 178 | 17.98 |  | ● | ● | ● |
| Chronic myeloid leukemia | 73 | 19.18 |  | ● |  |  |
| Colorectal cancer | 62 | 24.19 |  | ● |  |  |
| ERBB signaling pathway | 87 | 22.99 |  | ● |  |  |
| Focal adhesion | 201 | 12.44 |  | ● |  | ● |
| Glioma | 65 | 26.15 |  | ● |  |  |
| Huntingtons disease | 185 | 7.03 | ● |  |  |  |
| Long term potentiation | 70 | 31.43 |  | ● |  | ● |
| MAPK signaling pathway | 267 | 14.23 | ● | ● |  | ● |
| Melanoma | 71 | 21.13 |  | ● |  |  |
| Neuroactive ligand receptor interaction | 272 | 16.91 |  | ● |  | ● |
| Neurotrophin signaling pathway | 126 | 34.13 | ● | ● |  |  |
| Pathways in cancer | 328 | 10.06 |  | ● |  | ● |
| Prostate cancer | 89 | 17.98 |  | ● |  |  |
| T cell receptor signaling pathway | 108 | 19.44 |  | ● |  |  |
| VEGF signaling pathway | 76 | 23.68 |  | ● |  |  |
| Reactome |  |  |  |  |  |  |
| Activation of NMDA receptor upon glutamate binding and postsynaptic events | 36 | 44.44 |  | ● |  | ● |
| CREB phosphorylation through the activation of ras | 26 | 46.15 |  | ● |  |  |
| CREB phosphorylation through the activation of camkii | 16 | 68.75 |  | ● |  |  |
| Neuroransmitter receptor binding and downstream transmission in the postsynaptic cell | 84 | 20.24 |  | ● |  | ● |
| Post NMDA receptor activation events | 32 | 40.63 |  | ● |  | ● |
| Reactome p75NTR signals via NFKB | 13 | 23.08 | ● |  |  |  |
| Signalling by NGF | 215 | 17.21 |  | ● |  | ● |
| Transmission across chemical synapses | 130 | 20.77 |  | ● |  | ● |
| Trka signaling from the plasma membrane | 103 | 17.48 |  | ● |  | ● |
| GO term |  |  |  |  |  |  |
| Anatomical structure development | 1005 | 3.58 | ● |  | -- | -- |
| Cell cell signaling | 400 | 12.00 |  | ● | -- | -- |
| Cell surface receptor linked signal transduction | 622 | 9.49 |  | ● | -- | -- |
| G protein coupled receptor protein signaling pathway | 326 | 11.04 |  | ● |  | ● |
| Glutamate receptor activity | 20 | 70.00 |  | ● | ● | ● |
| Glutamate signaling pathway | 17 | 52.94 |  | ● | ● | ● |
| Growth factor activity | 54 | 7.41 | ● |  |  |  |
| Multicellular organismal development | 1040 | 3.56 | ● |  | -- | -- |
| Neurological system process | 377 | 9.81 |  | ● |  | ● |
| Nervous system development | 382 | 6.02 | ● |  | -- | -- |
| Receptor binding | 373 | 6.17 | ● |  |  | ● |
| Synaptic transmission | 172 | 18.60 |  | ● | ● | ● |
| System development | 855 | 3.86 | ● |  | -- | -- |
| Transmembrane receptor activity | 411 | 10.22 |  | ● | -- | -- |
| Transmission of nerve impulse | 187 | 17.11 |  | ● |  | ● |
| Chemical and genetic perturbations |  |  |  |  |  |  |
| Amit EGF response 120 HeLa | 68 | 2.94 | ● |  |  |  |
| Basaki YBX1 targets up | 293 | 0.68 | ● |  |  |  |
| Browne HCMV infection 10hr dn | 57 | 3.51 | ● |  |  | ● |
| Buytaert photodynamic therapy stress dn | 646 | 1.08 | ● |  | -- | -- |
| Charafe breast cancer basal vs mesenchymal dn | 51 | 1.96 | ● |  |  | ● |
| Charafe breast cancer luminal vs mesenchymal dn | 456 | 1.54 | ● |  | -- | -- |
| Creighton endocrine therapy resistance 3 | 723 | 0.41 | ● |  | -- | -- |
| Dacosta UV response via ERCC3 common dn | 420 | 3.10 | ● |  | -- | -- |
| Dacosta UV response via ERCC3 dn | 855 | 2.81 | ● |  | -- | -- |
| Dacosta UV response via ERCC3 XPCS dn | 75 | 2.67 | ● |  |  |  |
| Dang regulated by MYC dn | 243 | 4.94 | ● |  |  | ● |
| Gentile UV high dose dn | 245 | 2.04 | ● |  |  |  |
| Gentile UV response cluster d2 | 34 | 5.88 | ● |  |  |  |
| Han SATB1 targets dn | 331 | 1.81 | ● |  |  | ● |
| Han SATB1 targets up | 318 | 2.83 | ● |  |  |  |
| Hellebrekers silenced during tumor angiogenesis | 56 | 5.36 | ● |  |  | ● |
| Kaab heart atrium vs ventricle dn | 267 | 1.12 | ● |  |  | ● |
| Kang immortalized by tert up | 89 | 6.74 | ● |  |  |  |
| Kim WT1 targets 12hr dn | 218 | 3.21 | ● |  |  |  |
| Kim WT1 targets dn | 471 | 1.27 | ● |  | -- | -- |
| Lopes methylated in colon cancer dn | 26 | 15.38 | ● |  |  |  |
| Mccabe bound by HOXC6 | 461 | 0.87 | ● |  | -- | -- |
| Mishra carcinoma associated fibroblast dn | 24 | 8.33 | ● |  |  |  |
| Mitsiades response to aplidin up | 459 | 2.18 | ● |  | -- | -- |
| Nakamura tumor zone peripheral vs central up | 288 | 0.35 | ● |  |  |  |
| Nuytten EZH2 targets up | 974 | 1.95 | ● |  | -- | -- |
| Perez TP53 targets | 1191 | 1.18 | ● |  | -- | -- |
| Ren alveolar rhabdomyosarcoma dn | 409 | 1.71 | ● |  | -- | -- |
| Rodrigues NTN1 targets up | 17 | 5.88 | ● |  |  |  |
| Takeda targets of NUP98 HOXA9 fusion 8d up | 157 | 3.18 | ● |  |  | ● |
| Toyota targets of mir34b and mir34c | 460 | 0.43 | ● |  | -- | -- |
| Wang smarce1 targets dn | 355 | 2.54 | ● |  |  |  |
| Wilcox presponse to rogesterone dn | 66 | 3.03 | ● |  |  |  |
| Zhang response to IKK inhibitor and TNF dn | 103 | 0.97 | ● |  |  |  |
| Other sources |  |  |  |  |  |  |
| ST differentiation pathway in PC12 cells | 42 | 28.57 |  | ● |  |  |

^a^ The total number of genes in each pathway annotated by MsigDB. ^b^ Pathway was selected since it was related to BDNF. ^c^ Pathway was selected since it include more than 10 percent I-Genes. ^d^ Pathway was significant in at least one statistical way. --: Did not include in pathway analysis since the extreme pathway gene numbers
